# Epidemiological Characteristics of Hospitalized Burn Patients—A 10-Year Retrospective Study in a Major Burn Center in Serbia

**DOI:** 10.3390/life15010118

**Published:** 2025-01-17

**Authors:** Marina Stojanović, Milana Marinković, Milana Jurišić, Biljana Miličić, Milan Stojičić, Milan Jovanović, Jelena Jeremić, Nemanja Dimić, Svetlana Srećković, Irina Drača Cetušić, Marko Jović

**Affiliations:** 1 Faculty of Medicine, University of Belgrade, 11000 Belgrade, Serbia; 2 Center for Anesthesiology and Resuscitation, University Clinical Center of Serbia, 11000 Belgrade, Serbia; 3 Clinic for Burns, Plastic and Reconstructive Surgery, University Clinical Center of Serbia, 11000 Belgrade, Serbia; 4 Department of Medical Statistics and Informatics, School of Dental Medicine, University of Belgrade, 11000 Belgrade, Serbia; 5 Clinic for Anesthesiology, Reanimatology and Intensive Care, Clinical Centre Dragiša Mišović-Dedinje, 11000 Belgrade, Serbia; 6 Department of Anestesiology and Intensive Care, Institute for Mother and Child Health Care of Serbia Dr Vukan Čupić, 11000 Belgrade, Serbia

**Keywords:** burns, burn unit, burn-related mortality, burns in low-to-middle-income countries

## Abstract

Background: Many European countries’ epidemiological data on burns were analyzed. This research aimed to analyze the key epidemiological characteristics of hospitalized burn patients in Serbia’s major burn unit over 10 years, as well as to create the very first national epidemiological dataset with the basic requirements for future epidemiological studies. Methods: A retrospective cross-sectional study was conducted, and demographic, clinical, and burn characteristics, as well as predictors of mortality, were analyzed. Results: A total of 996 patients were included. The mean age of the population was 54.65 ± 27.15 years. Regarding etiology, flame was the most common (49.0%). Patient comorbidities were noted on admission in 50.1% of cases. The mean % of total burn surface area (TBSA) was 16.89 ± 18.72%. Inhalation injury was confirmed in 7.5% of patients, and a total of 10.3% of patients required mechanical ventilation during hospitalization. The requirement for mechanical ventilation support was the strongest independent predictor of mortality, while other independent predictors of mortality were male gender, higher %TBSA, deep burns, mechanism of injury, an extended total length of stay (LOS), the occurrence of complications during hospitalization, and conservative treatment modalities. Conclusion: Burn injury mortality remains high, and %TBSA, burn depth, mechanical ventilation requirement, inhalation injury, and the presence of comorbidities on admission unfavorably influence mortality rates.

## 1. Introduction

Burns continue to be a major source of severe morbidity and mortality, with significant health and economic consequences [1]. According to the World Health Organization (WHO), over 180,000 people die each year as a result of burn injuries [2,3]. Even though the treatment and prevention of burns has advanced significantly in the past 30 years, reducing morbidity and mortality globally, burn-related mortality remains high. The high cost of care for burn patients burdens healthcare systems, influencing patient outcomes in some countries [4,5,6,7,8,9,10].

Epidemiological data regarding burns from many developed European countries have been studied, providing reports of pooled data regarding the epidemiology of burns in Europe [4,5,6,7,8,9,10,11,12,13,14,15,16]. Yet, to the best of our knowledge, no studies have been published regarding the epidemiology of burns in Serbia. The objective of the present study is to investigate the essential epidemiological characteristics of hospitalized burn patients in the Burn Unit of the University Clinical Center of Serbia in Belgrade over 10 years. We aim to obtain initial national epidemiological data with basic requirements for further epidemiological studies.

## 2. Materials and Methods

The study was designed as a retrospective cross-sectional study. Data were collected from the medical records of patients with burn injuries treated at the Clinic for Burns, Plastic and Reconstructive Surgery of the University Clinical Center of Serbia in Belgrade from 2013 to 2023. This study conformed to the World Medical Association Declaration of Helsinki and was approved by the Institutional Review Board (229/24, 15 March 2024).

### 2.1. Baseline Description of the Burn Unit, Clinic for Burns, Plastic and Reconstructive Surgery, University Clinical Center of Serbia

Serbia has an estimated total population of 6,647,003 inhabitants with a moderate overall population density (5.8 inhabitants per square kilometer). By the latest data, Serbia is classified as an upper-middle-income country, with a human development index (HDI) of 0.802, placing it 63rd on the very-high HDI list of countries, as well as a Gini index of 35.0, measuring the distribution of income across a population in terms of inequality [17,18,19].

The Burn Unit of the Clinic for Burns, Plastic and Reconstructive Surgery, Clinical Center of Serbia, Belgrade, serves as the largest national burn center in Serbia for adult civilian patients, and it often admits extensively burned patients or complicated transfers of patients from other hospitals in the country. The burn unit is equipped with an intensive care unit (ICU) of 8 isolated beds and two semi-ICU rooms with 10 hospital beds. Other clinical centers in Serbia do not have specialized burn units, and burn patients are seldom treated in general intensive care units. Skin banks do not exist in Serbia at the moment, nor are the services of the Euro Skin Bank available to Serbia. Moreover, skin substitutes are not currently provided by national health insurance and are thus unavailable to most patients. Surgical treatment for these patients typically involves the auto- or homotransplantation of skin, the use of flaps, or, in the most severe cases, the amputation of limbs or parts of the body. For the treatment of extensive deep burns, the only viable option is the homotransplantation of skin, with voluntary skin donors being rare. The dressing of the donor sites generally involves the use of Vaseline, gauze, and antiseptics or hydrocolloid dressings, depending on the surgeon’s preferences.

Admissions to the burn unit are based on the European Burn Association (EBA) criteria: patients who require burn shock resuscitation; patients with partial-thickness burns affecting more than 20% of total burn surface area (TBSA) in adults under the age of 65 or 10% in adults over the age of 65; full-thickness burns of any extent; burns affecting the face, hands, genitalia, or major joints; circumferential burns; burns of any size complicated by severe medical disorders or trauma; suspected inhalation injury; electrical burns, including lightning injuries; chemical burns; and patients with burns who require social or emotional rehabilitation support [20]. Additional hospitalization criteria include burns eligible for outpatient care, deemed infected in terms of incipient phlegmon, or necessitating intravenous antibacterial therapy.

### 2.2. Patient Data and Selection Criteria

Patients of both sexes treated at the burn unit from 2013 to 2023 who met the hospitalization criteria were included. Patients under the age of 18, patients discharged against medical advice, and patients with bullous dermatoses were excluded from the study. All data were extracted retrospectively from patients’ medical records.

Demographic and clinical characteristics, including age, gender, comorbidities, TBSA, burn depth, and burn etiology, were collected. Comorbidities were divided into cardiovascular (including hypertension, angina pectoris, myocardial infarction, cardiomyopathy, arrhythmia, condition after myocardial revascularization, heart failure), psychiatric (including schizophrenia, depression, addiction, anxiety, bipolar disorder, suicide attempts), neurological (including dementia, condition after stroke, paralysis, paresis), pulmonary (including chronic obstructive pulmonary disease, pulmonary hypertension, atelectasis), renal (chronic renal failure, polycystic kidneys), gastrointestinal (inflammatory bowel diseases, liver diseases, presence of gastric or duodenal ulcer), and oncological diseases, as well as diabetes mellitus. Further, data regarding the time from injury to admission, total length of stay (LOS), presence of inhalation injury, necessity for mechanical ventilation assistance, and duration were collected and analyzed. Additionally, the treatment method, the time from injury to surgery, complications during treatment, and outcomes were examined.

### 2.3. Statistical Analysis

IBM SPSS software for Windows version 21.0 (Chicago, IL, USA 2021)was used for statistical data analysis. Numerical variables are shown in the form of mean values ± standard deviation and minimum and maximum values, while categorical variables are shown as absolute numbers and percentages. Using the Kolmogorov–Smirnov test, the normality of the data distribution was checked. Pearson’s χ^2^ test (contingency tables) was used to analyze data with a normal distribution, while the Mann–Whitney U test was used to compare differences between two groups where the dependent variable was ordinal or continuous without a normal distribution. Predictors of mortality were determined by logistic regression analysis. Values of *p* < 0.05 were considered statistically significant.

## 3. Results

### 3.1. Patient Demographics and Burn Etiology

A total of 996 patients met the inclusion criteria, with an annual hospitalization rate of 90 to 100 patients per year, equaling an admission incidence rate of 1.86/100,000 population/year. Overall, 70.4% of the included patients were male, while 29.6% were female. The mean age of the study population was 54.65 ± 27.15 years (ranging from 18 to 92 years). The most prevalent mechanism of injury was by flame, accounting for 49.0% of the cases, followed by scalding (22.2%), gas explosion (12.1%), electricity (9.4%), contact burns with hot objects (4.6%), and other causes such as chemical or unknown etiology (2.7%). The median time from injury to hospital admission was 8 h (interquartile range (IQR): 68.0). In 71.6% of patients, the injury occurred in the 48 h before hospital admission, while 28.4% of patients were admitted more than 48 h following injury. Patient comorbidities were noted on admission in 50.1% of cases, with cardiovascular diseases (32.3%) and diabetes mellitus (12.7%) being the most frequent. About 17.1% of patients had two or more chronic diseases. General demographics, patient admission data, and comorbidities on admission are shown in Table 1.

### 3.2. Burn Injury Characteristics and Management

Overall data regarding %TBSA, burn depth, mechanical ventilation, surgery, and complications are presented in Table 2. The mean %TBSA was 16.89 ± 18.72%. Superficial burns (IIa degree) accounted for 38.0% of total burns, while deep burns (IIb and III degree) accounted for 62.0%. About 60.6% of patients were treated conservatively, while 39.4% required surgery. Conservative treatment resulted in a greater mortality rate than surgical treatment (23.5% vs. 13.0%, *p* < 0.001). The median time from injury to surgery was 8 days (IQR 11). In patients who had a fatal outcome, operative treatment was started earlier than in patients who survived (*p* = 0.006). About 26.7% of cases required only one operation, while 0.1% of cases received as many as eight surgeries. The median length of stay was 15 days (IQR 18.0).

Inhalation injury was confirmed in 7.5% of patients, while a total of 10.3% of patients required mechanical ventilation during hospitalization. Mechanical ventilation was associated with a lethal outcome (47.4% vs. 1.4%, *p* < 0.001).

Complications occurred in 20.7% of patients. The most common complications included sepsis, pneumonia, acute renal failure, psychogenic syndrome, pleural effusions, and pulmonary thromboembolism (17.5%, 7.6%, 3.6%, 2.6%, 2.3%, and 2.0%, respectively). Complications were strongly associated with higher mortality, with sepsis, the occurrence of pneumonia, acute renal failure, pulmonary thromboembolism, and acute myocardial infarction having the strongest association with a lethal outcome (*p* < 0.001).

Table 3 shows factors contributing to mortality. The overall mortality rate of the cohort was 19.3%. Mortality was higher in patients injured in the first 48 h before admission compared to those admitted 48 h past injury (22.4% vs. 11.7%, *p* < 0.001). Mortality was significantly associated with male gender (62.5% men vs. 37.5% women, *p* = 0.011), age (63.66 ± 18.05 in deceased vs. 52.48 ± 28.51 in surviving patients, *p* < 0.001), a higher percentage of TBSA (41.24 ± 25.42% vs. 10.98 ± 10.03, *p* < 0.001), and burn depth (2.6% superficial vs. 29.5% deep burns, *p* < 0.001). Regarding burn mechanism, patients with injury by flame had the highest mortality (*p* < 0.001), followed by those injured by scalds, gas explosions, contact burns, and electrocution (27.7%, 21.0%, 13.9%, 13.0%, 6.5%). The presence of comorbidities significantly increased mortality (*p* < 0.001). Patients with cardiovascular diseases, diabetes mellitus, psychiatric diseases, and diseases of the respiratory system had significantly higher mortality (*p* < 0.001, *p* = 0.015, *p* < 0.001, *p* = 0.036, respectively), while neurological, renal, and gastrointestinal diseases did not affect mortality.

In the univariate logistic regression analysis, male gender, older age, the presence of comorbidities, %TBSA, and deep burns were associated with higher mortality, while an injury-to-admission time longer than 48 h was associated with a favorable outcome. The presence of inhalation injury, the requirement for mechanical ventilation support, early surgical excision, and the occurrence of complications were all associated with a fatal outcome. The results of the univariate logistic regression with *p* values are presented in Table 4.

In the multivariate logistic regression, the requirement for mechanical ventilation support was the strongest independent predictor of mortality. Other independent predictors of mortality included male gender, %TBSA, deep burns, mechanism of injury, an extended LOS, the occurrence of complications during hospitalization, and conservative treatment modalities. These results with corresponding *p* values are presented in Table 5.

## 4. Discussion

### 4.1. Incidences and Annual Number of Admissions

An average annual hospitalization rate between 90 and 100 patients per year was observed in the studied 10-year period at the Burn Unit of the Clinic for Burns, Plastic and Reconstructive Surgery, Clinical Center of Serbia. There is currently no national registry for burn patients in Serbia. Given the current organization of the healthcare system, there is no unified DRG system in place, making it impossible to determine the exact incidence of burn patients and number of admissions at this time. Based on the published material, the average number of admissions to burn units varies greatly by continent and country. Burn units in Australia record the highest number of annual admissions of patients (2000/year), followed by units in Asia and North America (501–1000/year) [16]. Central and South America, as well as Europe, have reported an annual admission rate of 101–250/year, while the lowest rates have been reported in Africa (50/year) [16]. While an overall estimate per continent provides useful insight into global burn injury trends, many factors influence the incidence of burn injuries and admission rates per country, most commonly the countries’ economies, education, and traditions. These factors differ significantly among low-income, low-to-middle-income, and high-income countries (LICs, LMICs, and HICs, respectively), influencing the per capita admission rates. In Europe, an admission rate of 17.0/100,000 population/year has been reported in Finland, 15.5/100,000 population/year in Norway, 4.66/100,000 population/year in the Netherlands, 18.9/100,000 population/year in Portugal, and 3.68/100,000 population/year in Spain [7,12,13,21,22]. Based on the 10 years from 2013 to 2023, the single-center admission rate to Serbia’s major burn center was 1.86/100,000 adult population/year. In comparison, for neighboring countries in the Balkan region, the most recent data on admission rates show 36.93/100,000 population/year reported in Romania and 5.2/100,000 population/year in Albania [5,21]. Reports of admission rates from other countries in the Balkan region are not currently available in the literature, and the provided admission rates include the pediatric population.

The latest data from one study estimated the age-standardized incidence of injuries related to fire, heat, and hot substances to be around 200/100,000 population in Serbia, as well as in Romania, and 198/100,000 population in Albania in 2017. In comparison, the age-standardized incidence was estimated to be 150/100,000 population in Norway and 159/100,000 population in Finland, while in the Netherlands, the age-standardized incidence reported was 84/100,000 population [23]. Thus, admission rates differ significantly among LICs, LMICs, and HICs, with some countries having a greater per capita incidence but, in most cases, a lower number of annual admissions [16]. These discrepancies between admission rates could be attributable to the financial capacity of healthcare systems, accessibility to medical services, and socioeconomic variances [15,24]. Data published in this study are some of the first regarding the epidemiology of burns in Serbia. They could serve as the foundation for establishing a national burn registry. Given that this is a single-center study of adult patients, the estimation of admission rates compared to the incidence of burn injuries in Serbia is limited and yet to be published.

### 4.2. Age, Gender, and Burn Etiology

The mean age of patients in this study was 54.65 ± 27.15 years, with the youngest patient being 18 and the oldest being 92 years old. Pediatric patients are referred to specialized burns units in children’s hospitals in Serbia; thus, patients <18 years were excluded from the study. There was no statistical difference in age between the genders. In the available literature, our results are consistent with the mean age of adult patients from other single-center studies in the Balkan region, Romania (mean age 55.8 ± 17.16 years), and Croatia (mean age 54.50 ± 20.21 years) [4,6].

In terms of the distribution of genders, men accounted for 70.4% of patients included in the study. Additionally, men were shown to have a greater mortality rate. These findings are consistent with the existing literature [7,8,16]. Globally, men account for approximately 56.38% of all burn patients worldwide, with some of the studies from Europe reporting 58.0% in Portugal, 61.9% in Spain, and 65.0% in Norway [7,12,16,25]. Similarly, in the studies from the Balkan region, 65.6%, 73.39%, and 61.3% male patients were reported in Romania, Croatia, and Albania, respectively [4,5,6]. Several causes are associated higher prevalence of burn injuries in men, such as occupational factors, outdoor activities, and behavioral factors [8,16,26]. Additionally, men have greater rates of drug and alcohol abuse, which may compromise perception and motor skills and increase the risk of accidents such as burns [1,27]. Furthermore, while men are generally more likely to work in high-risk occupations such as engineering, in LMICs, informal employment arrangements are still a common practice, often leaving employees more vulnerable to burns due to lack of implementation of occupational safety standards, limited utilization of personal protective equipment, and inadequate access to safety instructions and education, resulting in a lack of knowledge and preventive measures in high-risk industries.

### 4.3. Burn Characteristics and Etiology

The mean TBSA in our study was 16.89 ± 18.72%, with 62.0% of cases corresponding to deep (IIb and III) burns. Burn depth and greater TBSA were associated with higher mortality, as expected. In other European countries, smaller TBSA values have been reported, with 13.0 ± 14.5% in Germany, 8.3% in Spain, and 10.9% in the Netherlands [7,8,9]. These differences could be due to different admission criteria, hospital capacity, and resources among burn units in different economies [27]. In an Albanian study, the mean %TBSA in patient groups >20 years old was around 30%, with a frequency of 17% full-thickness burns in all patients, increasing in an age-related fashion, reaching 41.6% full-thickness burns in the age group of >80 years [5]. Regarding burn depth, our results were similar to those reported among Romanian adult patients, where deep burns occurred in 63.4% of cases [6].

In our study, flame was the most common cause of burns (49.0%). Additionally, direct exposure to flame was an independent predictor of mortality, in regard to the mechanism of injury. These findings are similar to those reported in burn units in Germany, the Netherlands, and Portugal, as well as Romania and Croatia [4,6,8,12,13]. Scalds represented the second most common mechanism of injury (22.2%), followed by gas explosions (12.1%). In the literature, the most frequently observed mechanism of injury in men was by flame, whereas scalding was the most common cause in women and children [7,8,28]. Additionally, in comparison to HICs, Gibson et al. found that LMICs reported a higher frequency of flame injuries (55.2 ± 1.4% in LMICs vs. 39.0 ± 0.9% in HICs), which could be attributed to occupational factors and insufficient safety protocols in LMICs, as described previously [28].

### 4.4. Comorbidities

Patients with preexisting health conditions are more likely to have extended hospital stays or unfavorable outcomes [1,9,29,30]. In our patient population, comorbidities were noted in 50.1% of patients. Patients with comorbidities had far higher mortality rates than individuals who were healthy prior to burn injury (70.8% vs. 29.2%, *p* < 0.001). Cardiovascular diseases, mental illnesses, diabetes mellitus, and respiratory system diseases were strongly correlated with a higher mortality. The mortality of patients with cardiovascular diseases was 47.9%, higher than in other studies [7]. Patients suffering from heart disease such as myocardial infarction or heart failure are known to have impaired hemodynamic stability, making it difficult to withstand the physiological stress of burns [29]. A patient’s cardiovascular, renal, pulmonary, or mental health issues may impair fluid resuscitation, while cardiovascular diseases alone compromise general perioperative and postoperative care [1,29]. Knowlin et al. reported that burn patients with preexisting heart diseases had a higher mortality rate than those without (16% vs. 3%) [29]. Arbaca et al. also found cardiovascular disease as a predictor of in-hospital mortality, increasing the probability of a lethal outcome by 112% in these patients [7].

Diabetes mellitus was the second most frequent comorbidity, with an 18.2% mortality rate in this patient sub-population. Diabetes is known to impair the immune system’s responsiveness, raising the risk of infection in burn wounds and urinary tract infections, as well as postoperative complications, while decreasing overall survival [1,31]. Additionally, Kim et al. reported that burn patients with diabetes more often required multiple surgical procedures [32]. Patients in our study had a higher incidence of preexisting health conditions compared to those in other European countries. Niculae et al. reported comorbidities in 44.1% of all patients hospitalized in a burn unit in Romania, while Abarca et al. noted 32.3% of patients with cardiovascular disease and 9.9% of patients with diabetes mellitus in a burn unit in Spain [6,7]. Colleagues from Croatia showed that patients with two or more comorbidities had 28-fold higher odds of a lethal outcome [4]. In our study, 17.0% of patients had two or more comorbidities on admission, significantly influencing mortality in the univariate regression model (OR 1.47, 95% CI 1.372–1.572, <0.001).

Patients with psychiatric disorders such as depression, schizophrenia, and anxiety disorders, as well as addiction, made up 8.8% of all included patients, with a mortality rate of 16.7% in this sub-population. According to the literature, patients with mental health disorders prior to admission had a higher TBSA and extended LOS, required multiple surgical procedures, and more often received prolonged mechanical ventilation [33]. While unintentional burn injuries remain the most prevalent mechanism of injury in psychiatric patients, suicide attempts by self-immolation present a special challenge. Mortality rates as high as 80% and prolonged hospitalization for self-immolation patients are described in the literature, with reports of 60–80% of patients having a previous psychiatric diagnosis [34,35]. Mannekote Thippaiah et al. reported the presence of suicidal intent as the main predicted risk factor for >40% TBSA burns in a recent publication [35].

A history of neurological, urological, or gastrointestinal illnesses had no significant effect on outcome lethality in the studied patients.

### 4.5. Treatment, Hospital Stay Length, Complications, and Outcome

The median time from injury to admission to our burn unit was 8 h (IQR 68.0), with the longest delay of 15 days (360 h). Overall, 28.4% of our patients had burns older than 48 h at the time of admission. Conversely, mortality rates were negatively associated with prolonged admission delays in surviving patients vs. delay in patients with lethal outcomes (*p* < 0.001). Given the importance of the first 48 h for hemodynamic stabilization, any delay in effective patient care could increase the likelihood of unfavorable outcomes. Still, many studies report no effect of the injury-to-admission time on mortality in burn patients, possibly due to the immediate referral of the most severely injured and critical patients to designated burn ICUs [36,37]. While injury-to-admission time did show a relationship with outcome in the univariate logistic regression in this study, in the multivariate logistic regression, it did not.

Surgery was performed in 39.4% of cases, while conservative treatment was applied for 60.6% of patients. In 26.7% of patients, only one surgery was sufficient, while in the most severe cases, up to eight operations were performed. The management of patients with extensive deep burns in Serbia presents significant challenges. This severe condition remains insufficiently recognized, and the available resources are extremely limited. According to established international guidelines for the treatment of such patients, skin substitutes are utilized, with surgical intervention commencing immediately after hemodynamic stability is achieved. As stated in the Methods section, skin banks do not exist in Serbia at the moment, nor are the services of the Euro Skin Bank available to Serbia. Thus, surgical management relies on skin auto-transplantation when applicable or skin homotransplantation from healthy volunteer donors, as well as the use of flaps.

Mortality rates in surgically managed patients were found to be lower than in conservatively managed patients (23.5% in the conservative treatment group vs. 13.0% in the surgical treatment group, *p* < 0.001). If we obtained access to a skin bank or skin substitutes, mortality would be probably reduced. The study did not focus on surgical techniques and their impact on mortality but only examined whether the patient underwent surgery or not. The median time from injury to surgery was 8 days (IQR 11), while, conversely, patients with a fatal outcome received surgical treatment earlier than those who survived (6 days (IQR 6) vs. 9 days (IQR 12)). Wong et al. analyzed the influence of early excision on mortality, sepsis occurrence, and LOS in a meta-analysis [25]. They demonstrated that though LOS was shorter and the incidence of sepsis was lower after performing early excision, mortality was lower when late excision was performed [38].

The median length of stay was 15 days (IQR 18.0). While the traditional 1 day of hospitalization per 1% TBSA provides a potentially adequate estimate of LOS for the management of patients’ and their loved ones’ expectations, many efforts have been invested in analyzing individual factors influencing LOS. Factors such as age, percentage of full-thickness burns, mechanism of injury (i.e., electrical injuries), days ventilated, and comorbidities such as mental illness, as well as complications, most commonly sepsis, are found to influence LOS. However, Dolp et al. reported in their retrospective cohort study that most of these non-modifiable factors are dependent on proper patient burn ICU care [39]. Thus, hospital resources and the number of beds, which depend greatly on the country’s income, play a significant role and should also be considered [8,16]. Opriessnig et al. reported that the average time spent in the ICU was between 11 and 14 days globally, while 22.15% of all burned patients spent more than 21 days in the ICU, with a notable difference between continents [16]. A mean ICU stay between 8 and 10 days was found in North America, 11–14 days in Central and South America, 15–20 days in Europe, and fewer than 3 days in Africa [16]. The mean time spent in the ICU also varied among European countries, with a mean ICU LOS of 13 days in Spain, 18 days in Germany, and 23 days in Romania [6,7,33]. In a Croatian study, the mean ICU LOS reported was 40.11 ± 37.65 days, with a LOS/TBSA% ratio in the surviving patients of 2.45 ± 1.5 [4]. In our study, a shorter LOS was associated with a favorable outcome, as expected, and corresponded to the median LOS in Europe.

Complications were observed in 20.7% of patients. The most common complication was sepsis, accounting for 17.5% of all the patients. The treatment of burn sepsis was carried out in a multidisciplinary manner. Anesthesiologists, plastic surgeons, internists, infectious disease specialists, and transfusion experts were all involved in the management of these patients. In addition to the appropriate treatment of the burn wound itself, antibiotic therapy played a crucial role. Antibiotic therapy started with the empirical administration of a broad-spectrum antibiotic until the results of the antibiogram were available. Once the results were obtained, the antibiotic regimen was adjusted if necessary. Depending on the clinical condition of the patient, symptomatic therapy was included, which, in the most severe cases, included various forms of respiratory support, inotropic stimulation, hemodialysis, and other interventions. Complications were an independent predictor of mortality, with sepsis being the main cause of death, which is similar to the rate of sepsis in Spain (16.3%) [17].The incidence of sepsis in burn patients with >20% TBSA ranges from 3% to 30% and is the most common cause of death. In comparison, data from the American Burn Association report multiorgan failure (MOF) being the main cause of death, with a 27.5% rate of lethal outcomes in burn patients [40,41]. Nonetheless, Kallinen et al. investigated causes of death in burn patients, reporting MOF as the cause of death in 40% of patients but noting that sepsis was always affiliated with multiorgan failure [42].

Mechanical ventilation was required in 10.3% of patients, while inhalation injury occurred in 7.5% of the patients and was significantly correlated with fatal outcomes in the univariate regression model (*p* < 0.001). In the multivariate regression model, the strongest independent predictor for mortality was the requirement for ventilatory support (OR 110.6, 95% CI 23.020–531.108). Abarca et al. reported that 9.4% of patients required mechanical ventilation in a Spanish cohort, while Niculae et al. found the frequency of inhalation injury in adult burn patients to be 40.9% and the need for mechanical ventilation 50.5% in a burn ICU in Romania [6,7]. While reports of mechanical ventilation vary in the literature, and intubation trends in burn patients have increased over the years, inhalation injury has been consistently reported as a poor prognostic factor in the literature [6,43,44,45].

In our study, 19.3% of patients had a lethal outcome. According to Opriessnig et al., global burn mortality was 18.27%. The greatest mortality has been reported in Africa (approximately 23.5%), and the lowest in North America (around 5.0%) [16]. In Europe, Spain reported a mortality of 4.3% in the adult population, similar to other developed European countries [7,25,33]. In the Balkans, Romania reported a mortality of 36.6% in a single-center study of adult patients, while in Albania, mortality ranged from 7.91% in the 15–60-year age group to 26.58% in the >60 age group [5,6].

This study’s primary limitation is its retrospective single-center design. Additionally, the limitations of this study are reflected in the absence of official national registries on injury and survival in burn patients and of a unified DRG within the entire country; thus, exact incidences, admission rates, and burn injury burden cannot be currently calculated. Nonetheless, to the best of our knowledge, this is the first study on the epidemiological characteristics of burn patients in Serbia, aiming to provide the basic requirements for further epidemiological studies.

## 5. Conclusions

In accordance with the existing literature, the factors associated with a higher risk of mortality were age, %TBSA, burn depth, and mechanical ventilation, as well as inhalation injury. Patients included in this study had higher rates of comorbidities on admission compared to other reports in the literature, unfavorably influencing mortality rates.

To the best of our knowledge, this is the first study on the epidemiological characteristics of burn patients in Serbia, providing essential data for further investigation and data pooling. Given the limited conditions such as the lack of skin substitutes and the unavailability of skin banks, we believe that conducting research on this issue holds national importance, with the aim of improving these resources in the future.

## Figures and Tables

**Table 1:** General demographics, patient admission data, and comorbidities at admission.

Variables	N = 996 (100%)
Age, (mean ± SD)	54.65 ± 27.15
Sex	
Male	701 (70.4%)
Female	295 (29.6%)
Mechanism of burn injury	
Flame	488 (49.0%)
Scalding	221 (22.2%)
Gas explosion	120 (12.1%)
Electricity	94 (9.4%)
Contact	46 (4.6%)
Other	27 (2.7%)
Injury-to-admission time (h), (median)	8 h (IQR 68.0)
Admissions in 48 h post-injury, n (%)	712 (71.6%)
Admissions after 48 h post-injury, n (%)	284 (28.4%)
Comorbidities n (%)	499 (50.1%)
Cardiovascular	322 (32.3%)
Diabetes mellitus	126 (12.7%)
Psychiatric	88 (8.8%)
Neurologic	66 (6.6%)
Pulmonary	14 (1.4%)
Gastrointestinal	10 (1%)
Oncological	6 (0.6%)
Renal	5 (0.5%)
Number of comorbidities	
0	497 (49.9%)
1	338 (34.0%)
2	132 (13.4%)
>3	37 (3.7%)

**Table 2:** Burn injury characteristics and treatment data.

Variables	N = 996 (100%)
TBSA%, (mean ± SD)	16.89 ± 18.72
Burn depth	
Superficial burns	375 (38.0%)
Deep burns	621 (62.0%)
Inhalation injury	74 (7.5%)
Type of treatment	
Surgery	392 (39.4%)
Conservative	601 (60.6%)
Number of surgeries	
0	601 (60.5%)
1	265 (26.7%)
2	76 (7.7%)
3	25 (2.5%)
4	19 (19.9%)
>5	8 (0.8%)
Injury-to-surgery delay (median)	8 days (IQR 11.0)
Mechanical ventilation, n (%)	102 (10.3%)
Length of stay (days), (median)	15 days (IQR 18.0)
Mortality, n (%)	192 (19.3%)
Complications, n (%)	208 (20.7%)
Sepsis	174 (17.5%)
Pneumonia	75 (7.6%)
Acute renal failure	36 (3.6%)
Psychoorganic syndrome	26 (2.6%)
Pleural effusion	23 (2.3%)
Pulmonary thromboembolism	20 (2.0%)
Acute myocardial infarction	8 (0.8%)
Cerebrovascular insults	5 (0.5%)
Surgical site infection	5 (0.5%)
Acute pancreatitis	3 (0.3%)

**Table 3:** Overall data on mortality rate.

Variable	Patients Who Survived N (%)	Patients with Lethal Outcomes N (%)	*p* Value
Sex			0.011
Male	579 (82.8%)	120 (17.2%)	
Female	223 (75.6%)	72 (24.4%)	
Age (mean ±SD)	52.48 ± 28.51	63.66 ± 18.05	<0.001
TBSA% (mean ±SD)	10.98 ± 10.03	41.25 ± 25.42	<0.001
Burn depth			<0.001
Superficial	365 (97.3%)	10 (2.7%)	
Deep	430 (70.3%)	182 (29.7%)	
Admissions within 48 h before injury	552 (77.6%)	159 (22.4%)	<0.001
Admissions over 48 h after injury	250 (88.3%)	33 (11.7%)	<0.001
Mechanism of injury			<0.001
Flame	352 (72.3%)	135 (27.7%)	
Scalds	124 (86.1%)	20 (13.9%)	
Gas explosions	100 (83.3%)	20 (16.7%)	
Electricity	87 (93.5%)	6 (6.5%)	
Contact	40 (87.0%)	6 (13.0%)	
Comorbidities	363 (45.3%)	136 (70.8%)	<0.001
Cardiovascular	230 (28.7%)	92 (47.9%)	<0.001
Diabetes mellitus	91 (11.3%)	35 (18.2%)	0.015
Neurologic	57 (7.1%)	9 (4.7%)	0.261
Psychiatric	56 (7.0%)	32 (16.7%)	<0.001
Pulmonary	8 (1.0%)	6 (3.1%)	0.036
Gastrointestinal	7 (0.9%)	3 (1.6%)	0.417
Renal	2 (0.2%)	3 (1.6%)	0.052
Number of comorbidities			<0.001
1	253 (31.5%)	85 (44.5%)	
2	88 (11.0%)	44 (23.0%)	
3	18 (2.2%)	9 (4.7%)	
4	6 (0.7%)	2 (1.0%)	
5	1 (0.1%)	1 (0.5%)	
Inhalation injury	23 (2.9%)	51 (26.7%)	<0.001
Mechanical ventilation	11 (1.4%)	91 (47.4%)	<0.001
Treatment			<0.001
Surgical	342 (42.6%)	51 (26.6%)	
Conservative	460 (57.4%)	141 (73.4%)	
Injury-to-surgery delay (median)	9 days (IQR 12)	6 days (IQR 6)	0.006

**Table 4:** Univariate regression analysis.

Variable	OR	95% CI	*p*
Sex	0.642	0.461–0.893	0.009
Age	1.023	1.014–1.032	<0.001
TBSA	1.106	1.090–1.123	<0.001
burn depth grade	2.404	1.909–3.029	<0.001
Superficial/deep burns	15.449	8.051–29.643	<0.001
Injury-to-admission time	0.996	0.994–0.998	<0.001
Admission within/over 48 h after injury	0.458	0.306–0.686	<0.001
Mechanism	0.719	0.645–0.801	<0.001
Comorbidities	2.937	2.089–4.130	<0.001
Number of comorbidities	1.750	1.476–2.073	<0.001
Length of stay	0.955	0.942–0.970	<0.001
Mechanical ventilation support requirement	64.789	33.519–125.233	<0.001
Duration of mechanical ventilation support	1.782	1.547–2.052	<0.001
Complications	1.468	1.372–1.572	<0.001
Inhalation injury	12.180	7.211–20.573	<0.001
Surgery treatment	0.486	3.343–0.690	<0.001
Number of surgeries	0.733	5.599–0.897	0.003
Injury-to-surgery delay	0.938	0.894–0.985	0.009

OR—odds ratio, CI—confidence interval, TBSA—total burned surface area.

**Table 5:** Multivariate regression analysis.

Variable	OR	95% CI	*p*
Sex	0.433	0.205–0.914	0.028
Age	1.006	1.000–1.013	0.059
%TBSA	1.102	1.069–1.136	<0.001
Burn depth	8.661	3.185–23.553	<0.001
Mechanism	0.747	0.590–0.946	0.015
Length of stay	0.938	0.913–0.963	<0.001
Mechanical ventilation	110.572	23.020–531.108	<0.001
Complications	1.592	1.375–1.844	<0.001
Surgical treatment	0.168	0.046–0.607	0.007

OR—odds ratio, CI—confidence interval, TBSA—total burned surface area.

## Data Availability

The data presented in this study are available on request from the corresponding author. The data are not publicly available due to Institutional Etics policy.
